# Development of mental disorders one year after exposure to psychosocial stressors; a cohort study in primary care patients with a physical complaint

**DOI:** 10.1186/1471-244X-12-120

**Published:** 2012-08-20

**Authors:** Lilli Herzig, Nicole Mühlemann, Bernard Burnand, Bernard Favrat, Nader Haftgoli, François Verdon, Thomas Bischoff, Paul Vaucher

**Affiliations:** 1 Institute of General Medicine, University of Lausanne, Rue du Bugnon 44, Lausanne 1011, Switzerland; 2 Department of Health and Community Medicine, University of Geneva, Michel-Servet 1, Geneva, 1211, Switzerland; 3 Institute of Social and Preventive Medicine, University of Lausanne, Route de la Corniche 2, Epalinges, 1066, Switzerland; 4 Department of Ambulatory Care and Community Medicine, University of Lausanne Rue du Bugnon 44, Lausanne, 1011, Switzerland

**Keywords:** Primary health care, Longitudinal studies, Mental disorder, Psychosocial deprivation, Stress

## Abstract

**Background:**

Mental disorders, common in primary care, are often associated with physical complaints. While exposure to psychosocial stressors and development or presence of principal mental disorders (i.e. depression, anxiety and somatoform disorders defined as multisomatoforme disorders) is commonly correlated, temporal association remains unproven. The study explores the onset of such disorders after exposure to psychosocial stressors in a cohort of primary care patients with at least one physical symptom.

**Method:**

The cohort study SODA (SOmatization, Depression and Anxiety) was conducted by 21 private-practice GPs and three fellow physicians in a Swiss academic primary care centre. GPs included patients via randomized daily identifiers. Depression, anxiety or somatoform disorders were identified by the full Patient Health Questionnaire (PHQ), a validated procedure to identify mental disorders based on DSM-IV criteria. The PHQ was also used to investigate exposure to psychosocial stressors (before the index consultation and during follow up) and the onset of principal mental disorders after one year of follow up.

**Results:**

From November 2004 to July 2005, 1020 patients were screened for inclusion. 627 were eligible and 482 completed the PHQ one year later and were included in the analysis (77%). At one year, prevalence of principal mental disorders was 30/153 (19.6% CI95% 13.6; 26.8) for those initially exposed to a major psychosocial stressor and 26/329 (7.9% CI95% 5.2; 11.4) for those not. Stronger association exists between psychosocial stressors and depression (RR = 2.4) or anxiety (RR = 3.5) than multisomatoforme disorders (RR = 1.8). Patients who are “bothered a lot” (subjective distress) by a stressor are therefore 2.5 times (CI95% 1.5; 4.0) more likely to experience a mental disorder at one year. A history of psychiatric comorbidities or psychological treatment was not a confounding factor for developing a principal mental disorder after exposure to psychosocial stressors.

**Conclusion:**

This primary care study shows that patients with physical complaints exposed to psychosocial stressors had a higher risk for developing mental disorders one year later. This temporal association opens the field for further research in preventive care for mental diseases in primary care patients.

## Background

Depression, anxiety and somatoform disorders are the most frequent mental disorders in primary care and the regularity of their occurrence is an important preoccupation in public health. WHO describes unipolar depression disorders as the fourth cause of burden of disease in the world and predicts they will rank second position in 2020 [1]. The World Mental Health Survey Initiative estimates that from 8.2-20.5% of Europeans has had at least one mental disorder during the previous year [2]. In addition, recent studies have shown the existence of an overlap between these disorders [3-5]. Many patients, at the onset of a mental disorder, first attend their general practitioner (GP) with a somatic complaint with or without underlying organic pathologies [6] putting the GP first in line to explore psychosocial distress as is expected by the patients [7,8].

Important efforts are being made to identify and understand the onset of mental disorders and the complex process leading from a simple disturbance to subthreshold and threshold illness [9]. Therefore many studies have explored different potential causal or contributory factors of mental disorders. Some of the factors identified have been gender [10,11], age [12,13], shaming experiences [13], major life events [14], chronic pain [15-17], chronic cardiac disease [18], socio-economic factors [19-23], early stress or childhood abuse [24,25], or other traumatism. Different psychosocial stressors such as stress at work or at home, financial problems or having no one to turn to when having problems are also described in association with mental disorders [5,26,27]. Different theoretical and biological explanations exist for this potential link [28,29]. However, epidemiological studies are lacking to support the causalities between psychosocial stressors and mental disorders [26,27]. The aim of this paper was therefore to explore the temporal association between the onsets of mental disorders after exposure to psychosocial stressors in a cohort of primary care patients each with at least one physical symptom.

## Methods

### Design

This cohort study was conducted by 21 GPs in private practice and three fellow physicians in an academic primary care centre located in the French-speaking part of Switzerland.

### Population

Eligible patients were: patients over 18 spontaneously reporting a physical complaint (new or recurrent). Exclusion criteria were: vital emergencies, home medical consultation, phone consultation, dementia, insufficient intellectual capacity (IQ under 65), inability to understand French and acute psychiatric disease.

The inclusion of all consecutive patients with physical complaints would have interfered with daily clinical practice; therefore each GP included one patient per half day of consultation selected by a randomized daily identifier. We assumed that 10–12 patients would be seen per half day of consultation and that half of those patients would exhibit a somatic symptom. Therefore, we prepared a series of lists containing rank orders of eligible patients: one of the ranking numbers was randomly determined to be the eligible patient of that half day. In the academic primary care centre, all consecutive eligible patients were asked to participate in the study as fewer patients were eligible.

### Case definition

Patients filled-out the French version of the full Patient Health Questionnaire (PHQ), a self-report version of the PRIME-MD, a validated procedure to identify mental disorders such as depression, anxiety and somatoform disorders [30-32]. The same criteria were used to identify cases at inclusion and after one year of follow-up and to exclude patients who were already affected by principal mental disorders at inclusion.

The construction of the PHQ, based on DSM IV criteria, allows providing provision diagnoses for a selection of DSM IV disorders. Therefore our criteria for defining depression and anxiety are based on the full DSM-IV criteria. For the diagnosis of somatoform disorder, we opted for the PHQ definition of multisomatoform disorders (MSD). MSD is defined by the presence of three or more unexplained physical complaints among 13 presented on a checklist, and by a history of chronic somatization [33]. MSD is detected by the PHQ questionnaire which is more relevant for the primary care setting than are DSM IV criteria. Physicians’ reported somatic diagnosis of their patients and we only considered somatoform symptoms if signs were not related to a reported diagnosis.

Focusing on the most frequent and overlapping mental disorders in primary care, we used the term “principal mental disorder” for any patients who suffered at least from depression or anxiety, or multisomatoform disorders.

### Exposure

We used the ten psychosocial stressors defined in question 12 of the PHQ. Participants were questioned on the subjective intensity of their exposure to these psychosocial stressors during the four weeks preceding the index visit. Major stressors are defined as those by which patients report being “bothered a lot” (subjective distress).

### Descriptive variables

The PHQ was completed with socio-demographic questions. “Higher education” was defined as university education or equivalent. GPs filled-out a separate questionnaire and collected data on comorbidities, consultation length, and diagnosis related to the chief complaint. GPs were blinded to the PHQ completed by patients at inclusion and at follow up.

### Data collection

Patients could either fill out their questionnaire at the GP’s office or post it later in a sealed envelope - these were transmitted each fortnight to the data centre. In addition, each patient was given a unique identification code and thereafter became anonymous. Each GP held a patient log-file to assure follow-up. The log was never transmitted to the data collection centre. The main diagnoses and comorbidities were coded according to a standardized pre-established coding list based on symptoms and suspected diagnosis. This list was established for coding purposes for ambulatory care in our Department of Ambulatory Care and Community Medicine, University of Lausanne, Switzerland.

### Follow-up

Patients were followed-up by their GPs as needed according to usual practice. The one year follow-up consultation took place during a scheduled visit 9–15 months after the index consultation. Patients who did not consult their physicians spontaneously during the one year follow-up were invited by phone to plan a visit within the next 3 months. A research nurse monitored recruitment and follow-up. Physicians were contacted to complete missing data.

### Statistical methods

We calculated a total of 616 patients to be included (relative risk of two, 7.5% of mental disorder in the control group versus 15% in the exposed group, level set at 0.05 and power at 0.8, 30% of the population having been exposed to a psychosocial stressor). Expecting 20% of the population to already be affected, the total number of patients to be questioned for inclusion was 740. The number of patients calculated to be required to assure statistical power for the objectives of a nested study was N = 1000.

Patients with depression, anxiety or MSD at baseline were not included in the analysis. Missing data were not replaced. Relative risk (RR) of developing a principal mental disease was first measured for each component (depression, anxiety, and MSD) before combining diseases together. RRs for developing a principal mental disorder were also first measured for each psychosocial stressor before combining them. Other factors associated both to psychosocial stressors and mental disease at a p-value of 0.2 or less using Fisher’s exact test were considered as potential confounding factors, but only if they were not considered as being on the causal pathway. Confounding effects were identified by comparing crude odds ratio (OR) with adjusted OR using logistic regression. Effect modification was evaluated by stratifying the analysis and testing homogeneity of RRs between groups using Mantel-Haenszel method. Significant level was set at 0.05. Linearity of association related to the number of exposed psychosocial stressors was tested by comparing logistic regression models with ordinal values versus dichotomized variables using the likelihood ratio test. Linearity was assumed if p>0.05.

### Consent and ethical approval

Patients were informed of the study and were included if they orally consented to participate. They explicitly acknowledged and consented to having their personal information sent to the data centre. The protocol was approved by the official Ethics Committee of the Canton of Vaud (Prot.100/04).

## Results

From November 2004 to July 2005, 1020 patients were screened for inclusion. 627 were eligible and 482 completed the PHQ at one year and were included in the analysis (77%). Median length of follow up was 14 months (ranging from 6–23 months).

**Figure 1:**
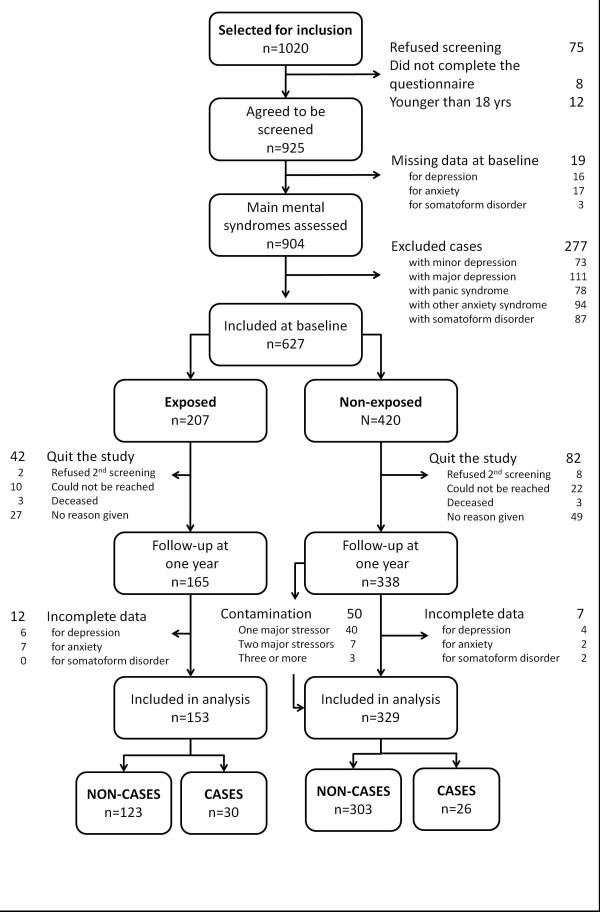
Flow chart.

Details on refusals, exclusions, drop-outs, missing data, and contamination are given in Figure 1. Patients already exposed to a psychosocial stressor were younger and more exposed to previous episodes of psychiatric disorders than other patients (Table 1). They were also more likely to suffer from psychiatric disorders other than depression, anxiety or MSD.

**Table 1:** Description of studied population

	**Exposed to major stressor**	**Not exposed**	**Fisher’s test**
	**n (%)**	**n (%)**	**p-value**
Age			P = 0.005
18 – 64.9 years	115 (75.2%)	204 (62.0%)	
≥ 65 years	38 (24.8%)	125 (38.0%)	
Gender			
Female	56 (36.6%)	132 (40.1%)	P = 0.484
Male	97 (63.4%)	197 (59.9%)	
Education			P = 0.577
Higher education	60 (39.2%)	110 (33.4%)	
Apprenticeship or equivalent	52 (34.0%)	119 (36.3%)	
Compulsory school or less	26 (17.0%)	57 (17.3%)	
Other or unknown	15 (9.8%)	43 (13.1%)	
Lives			P = 0.683
With family or friends	117 (76.5%)	244 (74.2%)	
Alone	29 (18.9%)	73 (22.3%)	
Unknown	7 (4.6%)	12 (3.5%)	
Occupation			P = 0.102
Full-time employment	45 (29.4%)	74 (22.5%)	P = 0.112
Part-time employment	28 (18.3%)	65 (19.8%)	P = 0.804
Housewife/-husband	28 (18.3%)	55 (16.7%)	P = 0.698
Retired	37 (24.2%)	114 (34.6%)	P = 0.026
Other	15 (9.8%)	21 (6.4%)	P = 0.200
Initial somatic complaint*			
Psychological origin	18 (11.8%)	22 (6.7%)	P = 0.075
Osteo-articular	52 (34.0%)	134 (40.7%)	P = 0.161
Neurological	9 (5.9%)	22 (6.7%)	P = 0.843
Cardiovascular	20 (13.1%)	28 (8.5%)	P = 0.141
Respiratory	16 (10.5%)	25 (7.6%)	P = 0.297
Digestive	17 (11.1%)	30 (9.1%)	P = 0.511
Uro-genital	7 (4.6%)	13 (3.9%)	P = 0.807
Mouth-throat	7 (4.5%)	27 (8.2%)	P = 0.182
Psychiatric condition			
Psychiatric comorbidities	34 (22.2%)	38 (11.5%)	P = 0.004
Psychological treatment in the previous 3 months	45 (29.4%)	67 (20.4%)	P = 0.037
Subjective health status†			P = 0.092
Excellent	11 (7.3%)	26 (7.9%)	
Very good	22 (14.6%)	81 (24.6%)	
Good	72 (47.7%)	147 (44.7%)	
Moderate	41 (27.1%)	68 (20.7%)	
Bad	5 (3.3%)	7 (2.1%)	

* Up to three diagnoses related to the complaint could be reported. Here we reports principle motive for common complaints only.

† Data was missing for two patients.

At one year, the prevalence of principal mental disorders was 30/153 (19.6%) for those initially exposed to a major psychosocial stressor and 26/329 (7.9%) for those who were not. In our analysis, we assumed that each stressor would be associated to a principal mental disorder in a similar way. To test this assumption, we measured the observed quantitative association for each stressor individually (Table 2). As the results do not suggest any differences in the strength of their association to one principal mental disorder, we were able to combine stressors together. We observed stronger associations between psychosocial stressors and depression or anxiety than with MSD (Table 3). Overall, patients with physical complaints who felt they were “bothered a lot” (subjective distress) by a psychosocial stressor were therefore 2.5 times (CI95% 1.5; 4.0) more likely to be affected by a mental disorder at one year than are other patients. From the 329 patients who had not already been exposed to a major psychosocial stressor, 50 reported having been exposed to a major psychological stressor during the follow-up period. This subgroup of patients represented the majority (17/26) of cases with acquired principal mental disorders in the control group.

**Table 2:** Exposure to major psychosocial stressors and the development of a mental disorder at one year (N = 482)

**Psychosocial stressors**	**Prevalence of mental disorders at one year**		**Relative risk of developing a mental disorder at one year**	
	**Exposed to stressor**	**Not exposed to stressor**	**RR (CI95%)**	**p-value**
	**% (n/N)**	**% (n/N)**		
Bothered a lot by				
Worrying about health	23.8% (10/42)	10.4% (46/440)	2.3 (1.2; 4.2)	0.001
Weight and appearance	24.2% (8/33)	10.7% (48/449)	2.3 (1.2; 4.4)	0.019
Little sexual desire or pleasure	20.0% (7/35)	11.0% (49/447)	1.8 (0.89; 3.7)	0.108
Difficulties with partner/lover	36.8% (7/19)	10.6% (49/463)	3.5 (1.8; 6.6)	0.0005
The stress of taking care of family members	5.3% (1/19)	11.9% (55/463)	0.44 (0.06; 3.0)	0.378
Stress at work or outside the home	20.7% (6/29)	11.0% (50/453)	1.9 (0.88; 4.0)	0.116
Financial problems or worries	15.8% (3/19)	11.4% (53/463)	1.4 (0.47; 4.0)	0.563
Having no one to turn to when having a problem	20.0% (2/10)	11.4% (54/472)	1.7 (0.5; 6.2)	0.403
Something bad that happened recently	17.1% (7/41)	11.1% (49/441)	1.5 (0.74; 3.2)	0.254
Thinking or dreaming about something terrible that happened in the past	27.8% (5/18)	11.0% (51/464)	2.5 (1.1; 5.6)	0.029
Number of major psychosocial stressors				
None		7.9% (26/329)	1	
One	15.0% (12/80)		1.9 (1.0;3.6)	
Two or more	24.7% (18/73)		3.1 (1.8; 5.4)	

**Table 3:** Relative risk of developing a mental disorder one year after initial exposure to at least one major psychosocial stressor

**Mental disorders**	**Prevalence of mental disorders at one year**		**Relative risk of developing a mental disorder at one year**	
	**Exposed to stressor**	**Not exposed to stressor**	**RR (CI95%)**	**p-value**
	**n = 153**	**n = 329**		
Depression				
Minor	10 (6.5%)	10 (3.0%)	2.2 (0.9; 5.1)	P = 0.073
Major	8 (5.2%)	6 (1.8%)	2.9 (1.0; 8.1)	P = 0.038
Either	18 (11.8%)	16 (4.9%)	2.4 (1.3; 4.6)	P = 0.006
Anxiety				
Panic disorder	7 (4.6%)	5 (1.5%)	3.0 (1.0; 9.3)	P = 0.045
Other anxiety	7 (4.6%)	4 (1.2%)	3.8 (1.1; 12.7)	P = 0.021
Either or both	13 (8.5%)	8 (2.4%)	3.5 (1.5; 8.3)	P = 0.002
Multi-somatoform disorders	9 (5.9%)	11 (3.3%)	1.8 (0.7; 4.2)	P = 0.193
Depression, anxiety or multi somatoform disorders	30 (19.6%)	26 (7.9%)	2.5 (1.5; 4.0)	P<0.001

We selected potential confounding factors by identifying all co-factors associated with psychosocial stressors (p<0.2 in Table 1) which were also associated with the development of principal mental disorders. These were: having moderate or bad health (RR = 2.1; 1.3 to 3.4; p = 0.004), and having undergone psychological treatment during the 3 previous months (RR = 1.8; CI95% 1.1 to 3.0; p = 0.019). Other co-factors were not significantly associated with principal mental disorders: 65 or older (RR = 0.78; CI95% 0.45 to 1.4; p = 0.377), in full-time employment (RR = 0.82; CI95% 0.5 to 1.4; p = 0.451), retired (RR = 0.88; CI95% 0.51 to 1.5; p = 0.636). Furthermore, experiencing pain assumed to be of psychological origin (RR = 1.1; CI95% 0.46 to 2.6; p = 0.856), of osteo-articular origin (RR = 0.82; CI95% 0.48 to 1.4; p = 0.446), of cardiovascular origin (RR = 0.89; CI95% 0.37 to 2.1; p = 0.784), of mouth-throat origin (RR = 0.51; CI95% 0.12 to 1.9; p = 0.279), and having psychiatric comorbidities (RR = 1.4; CI95% 0.76 to 2.6; p = 0.293) were also not significantly associated with principal mental disorders. The odds of developing a principal mental disorder decreased slightly from 2.8 (CI95% 1.6 to 5.0) to 2.6 (CI95% 1.5 to 4.7) after adjusting for subjective health status and previous psychological treatments. This suggests that the observed association between psychosocial stressors and principal mental disease is not confounded by any of the studied factors.

The linearity of the association of principal mental disorders to the number of exposures to major psychosocial stressors (0, 1, 2 or more; Table 3) can be assumed (Likelihood-ratio test p = 0.885). This linearity is maintained after adjusting for confounders (p = 0.780). Nevertheless, we cannot exclude ceiling effects to occur with increased number of stressor events.

We examined whether the relative risk of developing a principal mental disorder varied between subgroups of patients (Table 4). The observed differences were not statistically significant.

**Table 4:** Relative risk of developing a mental disorder when exposed to psychosocial stressors within subgroups

	**Stratified RR**	**Test of homogeneity**
	**OR (CI95%)**	**(*Mantel-Haenszel)***
Age		
Under 65	3.1 (1.7 to 5.6)	
65 and over	1.4 (0.5 to 3.8)	P = 0.249
Gender		
Male	4.3 (1.7 to 11.1)	
Female	1.9 (1.1 to 3.4)	P = 0.153
Psychiatric comorbidities*		
With	1.3 (0.4 to 4.0)	
Without	2.8 (1.6 to 4.8)	P = 0.238
Psychological treatment		
Last 3 months	1.5 (0.7 to 3.3)	
None during last three months	3.0 (1.6 to 5.6)	P = 0.165
Subjective health		
Good to excellent	2.9 (1.5 to 5.5)	
Moderate to bad	1.8 (0.9 to 3.7)	P = 0.325

*reported by physician at baseline.

## Discussion

Our study shows that the risk of suffering from principal mental disorders (depression, anxiety and multisomatoforme disorders) at one year is 2.5 times more likely when primary care patients with initial somatic complaints have been exposed to psychosocial stressors at the time of the index visit. This temporal association is one additional contributing element to the potential causal role of psychosocial stressors on the occurrence of frequent mental disorders [34]. The association is maintained independently of previous psychological treatment or subjectively perceived health-status. Furthermore, our study shows that the addition of different stressors increases the risk of developing principal mental disorders. Finally, the association between psychosocial stressors and multisomatoform disorders was weaker than the association with either depression or anxiety. Indeed multisomatoform disorders might be related to different etiologies and to different underlying neurobiological mechanisms.

### Strengths and limitations

The strength of this cohort study is its design, which allows analysis of the prospective association between exposure to psychosocial stressors and the onset of a principal mental disorder. Furthermore, the number of participating GPs and patients included are sufficient to be representative of the overall population in primary care in Switzerland. Finally, our study is focused on the onset of depression, anxiety or MSD in patient with at least one somatic complaints in PC; a subset of patients rarely seen or studied by specialists.

Our study has some limitations. First, the combination of depression, anxiety and MSD under the single term of “principal mental disorder” assumes that these elements share a common underlying biological mechanism, which is not necessarily true. However, the observed overlap between these three principal mental disorders in primary care can justify our choice [3,4]. Second, using the broader definition of MSD proposed by Kroenke et al. [33] instead of the full DSM IV criteria could have induced false positive diagnosis of somatoform disorder. Third, our case definition of depression, anxiety and multisomatoforme disorders was based on the PHQ. Even if the PHQ is considered as a relevant proxy for screening principal mental disorders in primary care [35,36], structured clinical interviews are considered as the gold standard to assess DSM-IV criteria for their diagnostic. Standardized interviews would have been challenging to organize, given that patients with somatic complaints are most often unwilling to visit a psychologist, mental social-worker, or a psychiatrist. Furthermore, in similar settings, the PHQ-9 showed an area under the receiver operating curve of 0.95 compared to structured clinical interviews [37,38] leading us to believe that for depression, results would have been similar had we used either of these diagnostic procedures. This might not have been the case for multisomatoform disorders for which different etiologies might be difficult to distinguish without a structured clinical interview. Fourth, it is not excluded that patients who develop physical complaints following a stress are also more likely to develop mental disorders. It is therefore uncertain that our observed association is as strong in patients without somatic complaint. Finally, psychosocial stressors might not all be associated to principal mental disorders. Some of them may also represent symptoms of psychiatric disorders. Our study cannot distinguish past exposure to psychosocial stressors from psychiatric symptoms that appeared before inclusion.

### Relation to other studies

Poleshuck et al. [26] studied the association between psychosocial stressors and depression for patients with musculoskeletal pain in a cross-sectional study. They found that depressive patients were more likely to report being bothered a lot by stressors (ORs ranging from 2.4 to 5.8) which was also described (ORs ranging from 2.0 to 4.5) in another cross-sectional study of our population [5]. In our longitudinal study, the strength of the association between psychosocial stressors and principal mental disorders was not as important (RR ranging from 0.44 to 3.5). This could be due to the fact that we excluded many exposed patients already affected at the time of inclusion.

Our results suggest that stressors might affect patients differently. However, none of these stressors were consistently more important in other studies [5,26]. We therefore believe most of these differences to be due to random error and small sample sizes. We nevertheless cannot exclude that different types of stressors might have different effects on the development of principal mental disorders.

Feeling “bothered” by an event could be due to the same factor that predisposes people to become depressive, anxious or develop MSD. Therefore, stressful events might not be at cause, but only the way people react to these events. Further animal behaviour studies are requested to analyse underlying mechanism linking stress to mental disorders to answer this question.

Overall, our observations are consistent with Bonde’s [27] systematic review of longitudinal studies of the effects of stressful conditions at work on depression (OR ranging from 1.4 to 2.3) in which different stressors were also combined. Recent research in neuroscience supports the belief that different stressors could have a similar effect on the onset of certain mental disorders [39]. This is also supported by the dose-dependent effect of stressors on the onset of disorders [5]. Clinically, questioning the patient’s personal interpretation and their subjective appreciation of different stressful life-events seems particularly important to the interpretation of their potential effects on their mental state [7,8].

Different psychological and social theories support a potential causal explanation for our results [9,22,40]. Physiological and behavioral consequences of social stress have been described in animal experiments. They show that mild stressors induce mild behavioral consequences but exposure to long-term or intensive stress has endocrinological effects (adrenal hypertrophy) [28]. Similar stress effects on humans may be one of the reasons for our results. Furthermore, Liston [29] describes the prefrontal consequences of stress, detected by pet scans and showing reversibility when stress is reduced.

The temporal association between psychosocial stressors and the onset of principal mental disorders, especially in patients with physical complaints, does incite GPs to explore these determinants in patients with somatic symptoms. Even if some mild principal mental disorders may resolve themselves [41,42] or at least not require formal medical or psychiatric help [8], more often they induce important human suffering, and increase health-care utilization and, therefore, health-care costs [43,44]. Also, it could be useful to detect risk factors early allowing preventive and treatment strategies.

It would be interesting to determine the relationships between exposure of certain stressor with the risk of developing a specifically disorder. But our study was not powered to test associations between stressors and mental disorders assuming each stressor had a similar effect on all three studied mental disorder. Given the sample size, it is not possible to verify this assumption.

## Conclusion

Mental disorders such as depression, anxiety and MSD are frequent in primary care, where they are often associated with physical symptoms and found in subthreshold states. Our study shows a 2.5 times increased frequency of the development of principal mental disorders in patients previously exposed to psychosocial stressors and is therefore an important step in highlighting the role of these stressors. Further studies are necessary to explore the weight of different stressors and if early screening and treatment strategies may diminish the onset of mental disorders.

## Ethical approval

The study protocol was approved by the official Ethics Committee of the Canton of Vaud (Prot.100/04).

## Abbreviations

CI: Confidence interval; DSM-IV: Diagnostic and Statistical Manual of Mental Disorder; GP: General practitioner; MSD: Multisomatoform disorder; OR: Odds ratio; PHQ: Patient health questionnaire; RR: Relative risk; SD: Standard deviation; SODA: Cohort study SOmatization, Depression, Anxiety.

## Competing interests

The authors declare that they have no competing interests.

## Authors’ contributions

LH conceived of the study, was principal investigator, participated in patients’ inclusion and wrote the manuscript. NM, NH, and TB participated in patients’ inclusion and critically revised the manuscript. BB and BF participated in the design of the study and the critical revision of the manuscript. FV participated in the design of the study, patients’ inclusion, and critically revised the manuscript. PV designed and performed the statistical analysis and co-wrote the manuscript. All authors accepted the manuscript after reading.

## Pre-publication history

The pre-publication history for this paper can be accessed here:

http://www.biomedcentral.com/1471-244X/12/120/prepub

## References

[B1] Mathers C Fat DM Boerma J The global burden of disease: 2004 update 2008 Geneva: World Health Organization

[B2] Demyttenaere K Bruffaerts R Posada-Villa J Gasquet I Kovess V Lepine JP Angermeyer MC Bernert S de Girolamo G Morosini P Prevalence, severity, and unmet need for treatment of mental disorders in the World Health Organization World Mental Health Surveys JAMA 2004 291 21 2581 2590 15173149 10.1001/jama.291.21.2581

[B3] Lowe B Spitzer RL Williams JB Mussell M Schellberg D Kroenke K Depression, anxiety and somatization in primary care: syndrome overlap and functional impairment Gen Hosp Psychiatry 2008 30 3 191 199 10.1016/j.genhosppsych.2008.01.001 18433651

[B4] Sartorius N Ustun TB Lecrubier Y Wittchen HU Depression comorbid with anxiety: results from the WHO study on psychological disorders in primary health care Br J Psychiatry Suppl 1996 30 38 43 8864147

[B5] Haftgoli N Favrat B Verdon F Vaucher P Bischoff T Burnand B Herzig L Patients presenting with somatic complaints in general practice: depression, anxiety and somatoform disorders are frequent and associated with psychosocial stressors BMC Fam Pract 2010 11 67 10.1186/1471-2296-11-67 20843358 PMC2945969

[B6] Kroenke K Spitzer RL Williams JB Linzer M Hahn SR deGruy FV Brody D Physical symptoms in primary care. Predictors of psychiatric disorders and functional impairment Arch Fam Med 1994 3 9 774 779 10.1001/archfami.3.9.774 7987511

[B7] Fritzsche K Armbruster U Hartmann A Wirsching M Psychosocial primary care - what patients expect from their General Practitioners A cross-sectional trial BMC Psychiatry 2002 2 5 10.1186/1471-244X-2-5 12000687 PMC113265

[B8] Walters K Buszewicz M Weich S King M Help-seeking preferences for psychological distress in primary care: effect of current mental state Br J Gen Pract 2008 58 555 694 698 10.3399/bjgp08X342174 18826781 PMC2553529

[B9] Harris T Recent developments in understanding the psychosocial aspects of depression Brit Med Bull 2001 57 17 32 10.1093/bmb/57.1.17 11719916

[B10] Borowsky SJ Rubenstein LV Meredith LS Camp P Jackson-Triche M Wells KB Who is at risk of nondetection of mental health problems in primary care? J Gen Intern Med 2000 15 6 381 388 10.1046/j.1525-1497.2000.12088.x 10886472 PMC1495467

[B11] Barnow S Linden M Lucht M Freyberger H-J The importance of psychosocial factors, gender, and severity of depression in distinguishing between adjustment and depressive disorders J Affect Disord 2002 72 1 71 78 10.1016/S0165-0327(01)00424-4 12204319

[B12] Arean PA Reynolds CF The impact of psychosocial factors on late-life depression Biol Psychiatry 2005 58 4 277 282 10.1016/j.biopsych.2005.03.037 16102545

[B13] Aslund C Nilsson KW Starrin B Sjoberg RL Shaming experiences and the association between adolescent depression and psychosocial risk factors Eur Child Adolesc Psychiatry 2007 16 5 298 304 10.1007/s00787-006-0564-1 17473948

[B14] Cohen S Janicki-Deverts D Miller GE Psychological stress and disease JAMA 2007 298 14 1685 1687 10.1001/jama.298.14.1685 17925521

[B15] Gureje O Simon GE Ustun TB Goldberg DP Somatization in cross-cultural perspective: a World Health Organization study in primary care Am J Psychiatry 1997 154 7 989 995 9210751 10.1176/ajp.154.7.989

[B16] Gureje O Von Korff M Simon GE Gater R Persistent pain and well-being: a World Health Organization Study in Primary Care JAMA 1998 280 2 147 151 10.1001/jama.280.2.147 9669787

[B17] Van Houdenhove B Psychosocial stress and chronic pain Eur J Pain 2000 4 3 225 228 10.1053/eujp.2000.0189 10985865

[B18] Frasure-Smith N Lesperance F Talajic M Depression following myocardial infarction. Impact on 6-month survival JAMA 1993 270 15 1819 1825 10.1001/jama.1993.03510150053029 8411525

[B19] Ihlebaek C Eriksen HR Occupational and social variation in subjective health complaints Occup Med (Lond) 2003 53 4 270 278 10.1093/occmed/kqg060 12815125

[B20] Weich S Lewis G Donmall R Mann A Somatic presentation of psychiatric morbidity in general practice Br J Gen Pract 1995 45 392 143 147 7772392 PMC1239175

[B21] Grassi L Rasconi G Pedriali A Corridoni A Bevilacqua M Social support and psychological distress in primary care attenders. Ferrara SIMG Group Psychother Psychosom 2000 69 2 95 100 10.1159/000012372 10671830

[B22] Lorant V Croux C Weich S Deliege D Mackenbach J Ansseau M Depression and socio-economic risk factors: 7-year longitudinal population study Br J Psychiatry 2007 190 293 298 10.1192/bjp.bp.105.020040 17401034

[B23] Weich S Lewis G Poverty, unemployment, and common mental disorders: population based cohort study BMJ 1998 317 7151 115 119 10.1136/bmj.317.7151.115 9657786 PMC28602

[B24] Stansfeld SA Clark C Caldwell T Rodgers B Power C Psychosocial work characteristics and anxiety and depressive disorders in midlife: the effects of prior psychological distress Occup Environ Med 2008 65 9 634 642 10.1136/oem.2007.036640 18388115

[B25] McCauley J Kern DE Kolodner K Dill L Schroeder AF DeChant HK Ryden J Derogatis LR Bass EB Clinical characteristics of women with a history of childhood abuse: unhealed wounds JAMA 1997 277 17 1362 1368 10.1001/jama.1997.03540410040028 9134941

[B26] Poleshuck EL Bair MJ Kroenke K Damush TM Tu W Wu J Krebs EE Giles DE Psychosocial stress and anxiety in musculoskeletal pain patients with and without depression Gen Hosp Psychiatry 2009 31 2 116 122 10.1016/j.genhosppsych.2008.10.003 19269531 PMC2677657

[B27] Bonde JP Psychosocial factors at work and risk of depression: a systematic review of the epidemiological evidence Occup Environ Med 2008 65 7 438 445 10.1136/oem.2007.038430 18417557

[B28] Zelena D Haller J Halasz J Makara GB Social stress of variable intensity: physiological and behavioral consequences Brain Res Bull 1999 48 3 297 302 10.1016/S0361-9230(98)00176-2 10229337

[B29] Liston C McEwen BS Casey BJ Psychosocial stress reversibly disrupts prefrontal processing and attentional control Proc Natl Acad Sci U S A 2009 106 3 912 917 10.1073/pnas.0807041106 19139412 PMC2621252

[B30] Spitzer RL Williams JB Kroenke K Linzer M deGruy FV Hahn SR Brody D Johnson JG Utility of a new procedure for diagnosing mental disorders in primary care. The PRIME-MD 1000 study JAMA 1994 272 22 1749 1756 10.1001/jama.1994.03520220043029 7966923

[B31] Spitzer RL Kroenke K Williams JB Validation and utility of a self-report version of PRIME-MD: the PHQ primary care study. Primary Care Evaluation of Mental Disorders. Patient Health Questionnaire. JAMA 1999 282 18 1737 1744 10.1001/jama.282.18.1737 10568646

[B32] Spitzer RL Williams JB Kroenke K Hornyak R McMurray J Validity and utility of the PRIME-MD patient health questionnaire in assessment of 3000 obstetric-gynecologic patients: the PRIME-MD Patient Health Questionnaire Obstetrics-Gynecology Study Am J Obstet Gynecol 2000 183 3 759 769 10.1067/mob.2000.106580 10992206

[B33] Kroenke K Spitzer RL deGruy FV Hahn SR Linzer M Williams JB Brody D Davies M Multisomatoform disorder. An alternative to undifferentiated somatoform disorder for the somatizing patient in primary care Arch Gen Psychiatry 1997 54 4 352 358 10.1001/archpsyc.1997.01830160080011 9107152

[B34] Hill AB The Environment and Disease: Association or Causation? Proc R Soc Med 1965 58 295 300 14283879 10.1177/003591576505800503 PMC1898525

[B35] Wittkampf KA Naeije L Schene AH Huyser J van Weert HC Diagnostic accuracy of the mood module of the Patient Health Questionnaire: a systematic review Gen Hosp Psychiatry 2007 29 5 388 395 10.1016/j.genhosppsych.2007.06.004 17888804

[B36] Wittkampf K van Ravesteijn H Baas K van de Hoogen H Schene A Bindels P Lucassen P van de Lisdonk E van Weert H The accuracy of Patient Health Questionnaire-9 in detecting depression and measuring depression severity in high-risk groups in primary care Gen Hosp Psychiatry 2009 31 5 451 459 10.1016/j.genhosppsych.2009.06.001 19703639

[B37] Kroenke K Spitzer RL Williams JB The PHQ-9: validity of a brief depression severity measure J Gen Intern Med 2001 16 9 606 613 10.1046/j.1525-1497.2001.016009606.x 11556941 PMC1495268

[B38] Kroenke K Spitzer RL Williams JB The PHQ-15: validity of a new measure for evaluating the severity of somatic symptoms Psychosom Med 2002 64 2 258 266 11914441 10.1097/00006842-200203000-00008

[B39] McEwen BS Gianaros PJ Central role of the brain in stress and adaptation: links to socioeconomic status, health, and disease Ann N Y Acad Sci 2010 1186 190 222 10.1111/j.1749-6632.2009.05331.x 20201874 PMC2864527

[B40] Bebbington PE Psychosocial causes of depression J Gend Specif Med 1999 2 6 52 60 11279872

[B41] Simon GE Goldberg D Tiemens BG Ustun TB Outcomes of recognized and unrecognized depression in an international primary care study Gen Hosp Psychiatry 1999 21 2 97 105 10.1016/S0163-8343(98)00072-3 10228889

[B42] Pini S Perkonnig A Tansella M Wittchen HU Psich D Prevalence and 12-month outcome of threshold and subthreshold mental disorders in primary care J Affect Disord 1999 56 1 37 48 10.1016/S0165-0327(99)00141-X 10626778

[B43] Katon W Sullivan M Walker E Medical symptoms without identified pathology: relationship to psychiatric disorders, childhood and adult trauma, and personality traits Ann Intern Med 2001 134 9 Pt 2 917 925 11346329 10.7326/0003-4819-134-9_part_2-200105011-00017

[B44] Valenstein M Vijan S Zeber JE Boehm K Buttar A The cost-utility of screening for depression in primary care Ann Intern Med 2001 134 5 345 360 11242495 10.7326/0003-4819-134-5-200103060-00007

